# PPE51 mediates uptake of trehalose across the mycomembrane of *Mycobacterium tuberculosis*

**DOI:** 10.1038/s41598-022-06109-7

**Published:** 2022-02-08

**Authors:** Mohammed Rizwan Babu Sait, Hendrik Koliwer-Brandl, Jessica A. Stewart, Benjamin M. Swarts, Marc Jacobsen, Thomas R. Ioerger, Rainer Kalscheuer

**Affiliations:** 1 Institute of Pharmaceutical Biology and Biotechnology, Heinrich Heine University, 40225 Düsseldorf, Germany; 2 Department of Chemistry and Biochemistry, Central Michigan University, Mount Pleasant, MI 48859 USA; 3 Department of General Pediatrics, Neonatology, and Pediatric Cardiology, University Children’s Hospital, Heinrich Heine University, 40225 Düsseldorf, Germany; 4 Department of Computer Science, Texas A&M University, College Station, TX 77843 USA

**Keywords:** Infection, Bacteriology, Microbial genetics, Pathogens

## Abstract

The disaccharide trehalose is essential for viability of *Mycobacterium tuberculosis*, which synthesizes trehalose de novo but can also utilize exogenous trehalose. The mycobacterial cell wall encompasses two permeability barriers, the cytoplasmic membrane and the outer mycolic acid-containing mycomembrane. The ABC transporter LpqY–SugA–SugB–SugC has previously been demonstrated to mediate the specific uptake of trehalose across the cytoplasmic membrane. However, it is still unclear how the transport of trehalose molecules across the mycomembrane is mediated. In this study, we harnessed the antimycobacterial activity of the analogue 6-azido trehalose to select for spontaneous resistant *M. tuberculosis* mutants in a merodiploid strain harbouring two LpqY–SugA–SugB–SugC copies. Mutations mediating resistance to 6-azido trehalose mapped to the proline–proline–glutamate (PPE) family member PPE51 (Rv3136), which has recently been shown to be an integral mycomembrane protein involved in uptake of low-molecular weight compounds. A site-specific *ppe51* gene deletion mutant of *M. tuberculosis* was unable to grow on trehalose as the sole carbon source. Furthermore, bioorthogonal labelling of the *M. tuberculosis* Δ*ppe51* mutant incubated with 6-azido trehalose corroborated the impaired internalization. Taken together, the results indicate that the transport of trehalose and trehalose analogues across the mycomembrane of *M. tuberculosis* is exclusively mediated by PPE51.

## Introduction

Tuberculosis (TB) is among the major infectious diseases that affects several million people every year. According to the World Health Organization TB Report 2019, there are about 1.5 million deaths every year due to TB^1^. TB is caused by *Mycobacterium tuberculosis (Mtb)*, a human pathogen that belongs to the class of actinobacteria^2^. The *Mtb* cell envelope consists of (from inside to outside) the cytoplasmic membrane, the mycolyl-arabinogalactan-peptidoglycan layer (mAGP), and a capsular layer mainly comprising α-glucan polysaccharides. The envelope confers physical robustness and protection against physicochemical stress and is also important for the virulence of the bacteria^3^. The mycolic acids are long chain α-branched β-hydroxy fatty acids that are either covalently bound to arabinogalactan, which in turn is linked to peptidoglycan, or esterified to sugars such as trehalose to give rise to the glycolipids, trehalose monomycolates (TMM) or trehalose dimycolates (TDM)^3^. Many components in the biosynthetic pathways of mycolic acids represent lucrative drug targets. TDM, also known as cord factor, is essential for the growth and survival of *Mtb*^4^. TDM provides virulence, stimulates host immune responses, and contributes to the interception of phagosomal maturation inside macrophages^5^. The arabinogalactan-bound mycolates form the inner leaflet and the trehalose mycolates form the outer leaflet of a lipid bilayer-like structure known as the mycomembrane that bears structural resemblance to the outer membrane of Gram-negative bacteria. The mycomembrane represents an efficient permeability barrier that contributes to the high intrinsic resistance of *Mtb* towards many antibiotics^6^.

Trehalose is crucial for the composition of mycobacterial cell envelope. De novo biosynthesis of trehalose in mycobacteria occurs through the OtsA–OtsB2 and the TreY–TreZ pathway^7–9^. TMM is synthesized in the cytoplasm by 6-*O*-mycoloylation of trehalose catalysed by Pks13 and is then transported to the mycomembrane through the mycobacterial membrane protein large 3 (MmpL3) transporter^10^. In the mycomembrane, the mycolic acid moiety from one TMM molecule is either transferred to another TMM molecule forming TDM, or it is transferred to arabinogalactan polysaccharides forming arabinogalactan-linked mycolates. These transfers are catalysed by the Antigen 85 (Ag85A, Ag85B, and Ag85C) complex leading to the concomitant release of free trehalose^3^. The free trehalose molecules are then recycled back to the cytoplasm through the LpqY–SugA–SugB–SugC ABC transporter located in the cytoplasmic membrane^11^. In addition to intrinsic de novo formation, *Mtb* can also utilize exogenous trehalose. Some flexibility regarding substrate specificity in the enzymatic machinery involved in trehalose uptake and metabolism also allows internalization of several synthetically altered trehalose analogues in a LpqY–SugA–SugB–SugC dependent manner and their subsequent metabolism and incorporation into the mycomembrane. These altered trehalose analogues might harbor structural modifications that allow conjugation with fluorescence probes employing biorthogonal chemistry^12,13^. However, while the LpqY–SugA–SugB–SugC ABC transporter exclusively mediates the uptake of trehalose across the cytoplasmic membrane, the route of trehalose transport across the mycomembrane is still unclear.

In this study, we harnessed the growth inhibitory properties of 6-azido trehalose (6-TreAz), which has anti-mycobacterial activity at high concentration^14^, for the isolation of spontaneously resistant mutants to identify genes potentially involved in trehalose uptake and its metabolism. This led to the identification of a member of the proline-proline-glutamate (PPE) family proteins. The *Mtb* genome encodes a high number of proteins comprising characteristic conserved proline-glutamate (PE) and PPE repeat motifs in their N-terminal region. There are 99 PE proteins and 69 PPE proteins encoded in the genome of the laboratory strain of *Mtb* H37Rv. PE/PPE proteins have been reported to be involved in modulation of the host immune response and in interaction with other bacteria^15,16^. Many PE/PPE proteins are secreted or cell surface-exposed and are substrates for type VII secretion systems^17,18^, with which they have co-evolved^19^. Some studies have previously suggested a role of certain PE/PPE proteins in nutrient uptake in *Mtb*^20^. Very recently, it has been shown that PPE51 is an essential integral mycomembrane protein responsible for transport of nutrients such as glucose, maltose and glycerol, as well for certain low-molecular-weight experimental drugs across the mycomembrane^21,22^. In the present study, we investigated the mechanism of trehalose uptake in *Mtb* in an unbiased approach and discovered a role in this process of the PPE51 mycomembrane transporter.

## Results

### 6-Azido trehalose inhibits *Mtb* growth at higher concentrations

**Figure 1:**
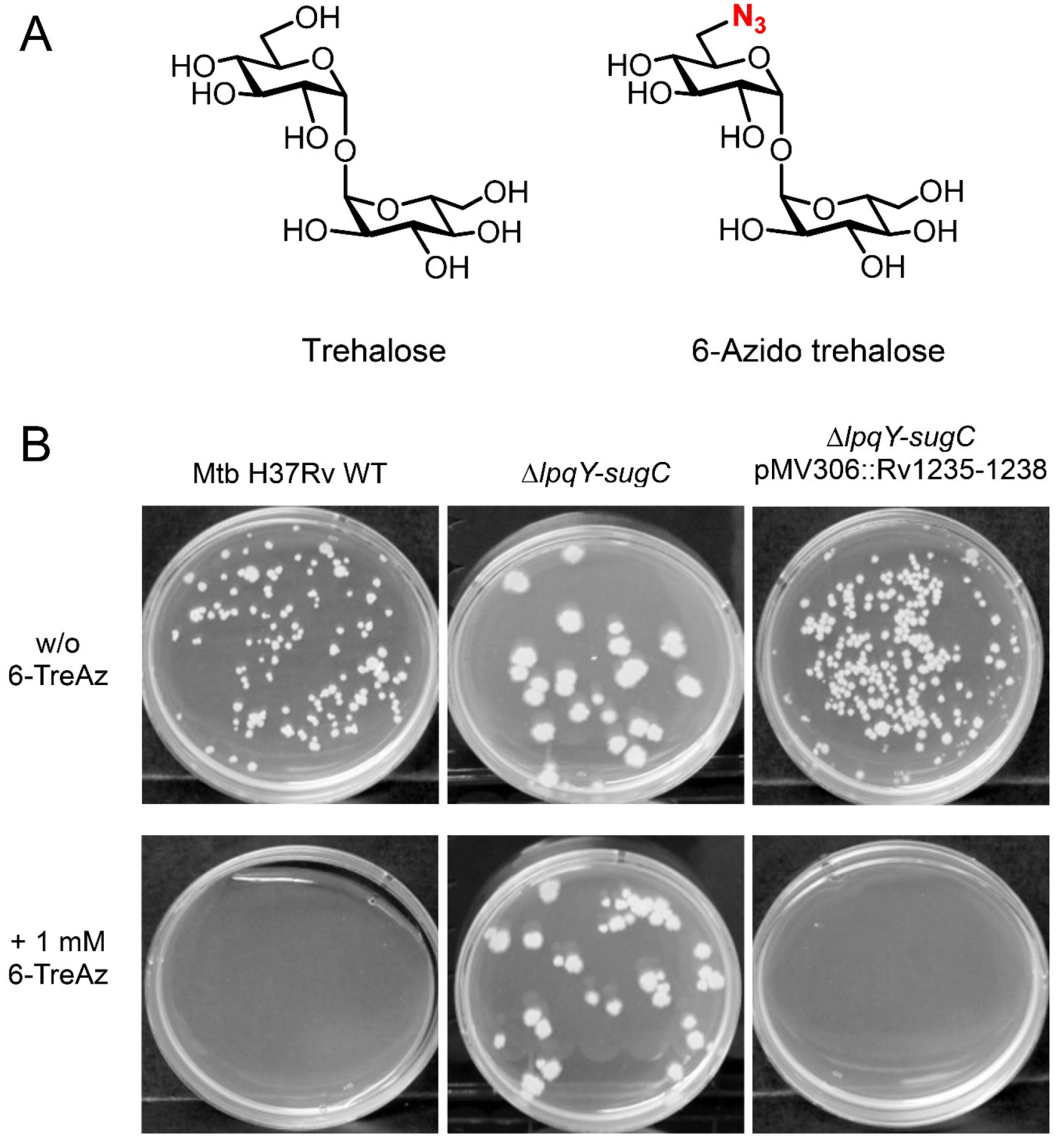
6-Azido trehalose (6-TreAz) inhibits *Mtb* growth at higher concentrations. (**A**) Structure of trehalose and 6-TreAz. (**B**) Growth inhibition of *Mtb* H37Rv wild-type (WT), the Δ*lpqY-sugC* mutant and the complemented mutant *ΔlpqY-sugC* pMV306(Kan)::Rv1235–1238 on Middlebrook 7H10 agar containing 1 mM 6-TreAz or just solvent control (1% DMSO = w/o 6-TreAz). Equal amounts of diluted cell suspensions of each strain were plated onto both types of agar and incubated for 3 weeks at 37 °C.

TMM is produced by 6-*O*-mycolylation of trehalose in the cytoplasm and is transported to the mycomembrane, where it is used to generate TDM with release of free trehalose. Differently modified trehalose analogs can also be taken up by *Mtb* and can undergo mycolylation. These modified TDMs and TMMs can be metabolically incorporated into the mycomembrane^12,13^. Modifications can occur at different positions of the trehalose molecule. 6-Azido trehalose (6-TreAz) has an azide group (N_3_) at the sixth carbon position of one of the glucose moieties in trehalose and has been used to label *Mtb* species (Fig. 1A). At concentrations ranging from 25 to 150 µM, 6-TreAz can be used as a bioorthogonal marker to label mycobacterial cells after conjugation with fluorescent moieties via click chemistry^13^. However, when incubated with increased concentrations, i.e. > 1 mM, we found that it inhibits growth of *Mtb* wild-type strain H37Rv on 7H10 solid medium. To further analyze this growth inhibition, we also tested the *Mtb* Δ*lpqY-sugC* mutant that lacks the complete LpqY–SugA–SugB–SugC (Rv1235–1238) ABC transporter, which recycles back the free trehalose that is produced by the antigen 85 complex when using TMM as the substrate^11^. The Δ*lpqY-sugC* deletion strain was able to grow on 7H10 solid medium containing 1 mM 6-TreAz (Fig. 1B). This highlights that the antibacterial effect of 6-TreAz is uptake-dependent and that LpqY–SugA–SugB–SugC not only tolerates trehalose but also 6-TreAz as a substrate. The growth inhibition of wild-type strain H37Rv by 6-TreAz likely results from the depletion of TDM in the mycomembrane as *Mtb* could not conjugate an additional mycolic acid to TMM because the 6′ position is occupied by the azide group, as already hypothesized previously for *Mycobacterium smegmatis*^23^. A previous study suggested that 6-TreAz also inhibits the trehalose synthase TreS when *Mtb* is grown under nutrient-limited biofilm conditions^24^. However, since TreS has been shown to be fully dispensable for growth of *Mtb* under nutrient-proficient in vitro culture conditions^8,25,26^, it is unlikely that potential inhibition of TreS contributes relevantly to the growth inhibitory effect of 6-TreAz as observed in our study.

### Spontaneous resistant mutants reveal a candidate responsible for extracellular trehalose uptake in *Mtb*

**Figure 2:**
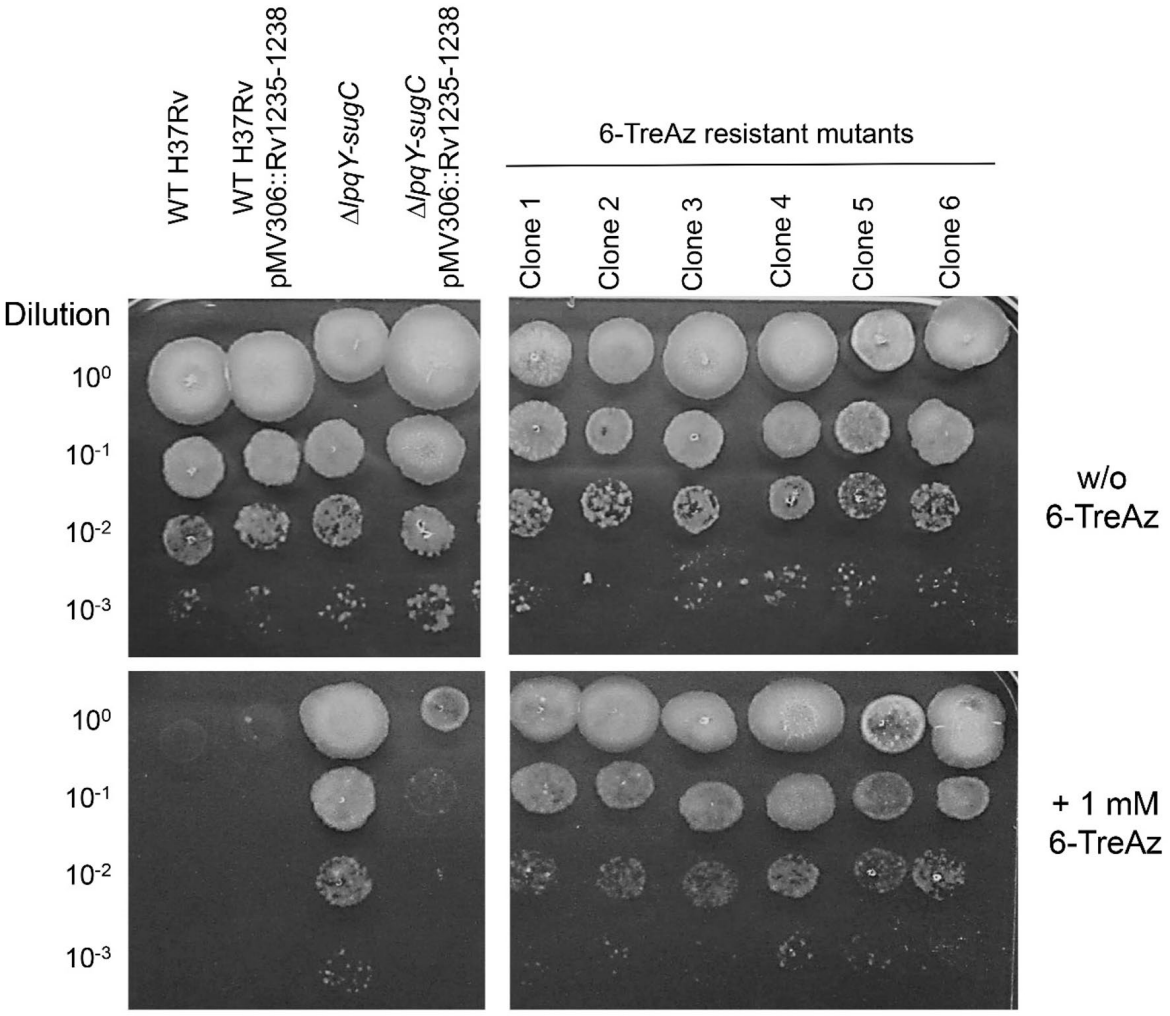
Isolation of spontaneous 6-TreAz-resistant *Mtb* mutants. 10 µl aliquots of tenfold-serially diluted cell suspensions of *Mtb* H37Rv wild-type (WT), the merodiploid strain *Mtb* H37Rv pMV306::Rv1235–1238, the Δ*lpqY-sugC* gene deletion mutant, the complemented mutant Δ*lpqY-sugC* pMV306::Rv1235–1238 and spontaneous 6-TreAz-resistant mutant clones were spotted each onto Middlebrook 7H10 agar containing 1 mM 6-TreAz or just solvent control (1% DMSO = w/o 6-TreAz). Plates were incubated for 3 weeks at 37 °C. For genotypes of mutant clones 1–4 and 6 refer to Table 1.

In order to identify genes potentially involved in transport and metabolism of trehalose in *Mtb*, we isolated spontaneous resistant mutants after growth on 6-TreAz-containing solid medium. In order to avoid isolating clones with loss-of-function mutations in the LpqY–SugA–SugB–SugC ABC transporter, which we have already shown to mediate 6-TreAz resistance, we employed a merodiploid strain that contains an additional copy of the *lpqY-sugC* gene cluster (Rv1235–1238) in its genome, which was generated by electroporating the integrative plasmid pMV306(Kan)::Rv1235–1238 into wild-type H37Rv (Supplementary Fig. 1). This additional copy of the gene cluster would compensate for any loss-of-function mutations in the endogenous locus and will minimize the chance of resistant mutants occurring from mutations in this gene cluster. After 4 weeks of incubation, 6-TreAz spontaneous resistant mutants were obtained at a frequency of 10^–7^ (Fig. 2). Whole genome sequencing of five selected clones revealed that independent non-synonymous mutations in *ppe51* (Rv3136) have occurred in three 6-TreAz-resistant mutants (nucleotide 661T→C, Leu204Pro; 41 G→C, Arg14Pro; 280 G→A, Ala94Thr). The two other spontaneous resistant clones harboured a non-synonymous mutation in the *eccC5* gene (Rv1783) (nucleotide 3278 C→A, Pro1093Gln). All clones except one *ppe51* mutant harboured additional mutations in different genes involved in phthiocerol dimycocerosate (PDIM) biosynthesis (*ppsA*, *ppsB*, *fadD26*; Table 1). We also generated complemented strains for some of these spontaneous resistant mutants constitutively expressing wild-type copies of *eccC5* or *ppe51*, respectively, from an integrative single-copy plasmid to check for their resistance on medium containing 6-TreAz, and we observed that the sensitivity of the resistant mutants was at least partially restored (Supplementary Fig. 2). These results indicate that the observed resistance phenotype could be clearly attributed to *eccC5* or *ppe51*, respectively. PPE protein family members have been shown to participate in uptake of nutrients through *Mtb* cell envelope, and PPE51 has been referred to as a mycomembrane-associated protein^27^. From previous studies it has been shown that EccC5, an ATP-binding protein belonging to the FtsK/SpoIIIE-like protein family, is required for secretion of ESX-5 specific substrates^28^. Further, it was shown that numerous PE and PPE proteins are secreted in an ESX-5-dependent manner in *Mycobacterium marinum*^18^. These findings suggest that PPE51 could be a potential gateway for the uptake of exogenous trehalose across the mycomembrane. The observed mutation in EccC5 might impair the ESX-5-mediated secretion of PPE51 and/or its proper translocation and insertion into the mycomembrane.

**Table 1:** List of spontaneous 6-TreAz-resistant mutants and identified gene mutations.

Mutant	Mutated gene	Nucleotide change	Amino acid change (total length of protein)	Other mutations (nucleotide change, amino acid change, total length of protein)
Clone 1	Rv1783c (*eccC5*)	3278 C→A;	Pro1093Gln (1391 aa)	*ppsA* (3882 G→A; Trp1294Stop; 1876 aa)
Clone 2	Rv1783c (*eccC5*)	3278 C→A;	Pro1093Gln (1391 aa)	*ppsA* (3882 G→A; Trp1294Stop; 1876 aa)
Clone 3	Rv3136 (*ppe51*)	661T→C	Leu204Pro (380 aa)	–
Clone 4	Rv3136 (*ppe51*)	41 G→C	Arg14Pro (380 aa)	*fadD26* (154 del C; Pro52fs; 583 aa)
Clone 6	Rv3136 (*ppe51*)	280 G→A	Ala94Thr (380 aa)	*ppsB* (38 del C; Thr13fs; 1538 aa)

Parental strain was merodiploid *Mtb* H37Rv pMV361::Rv125–1238.

*fs* frame-shift, *del* nucleotide deletion.

### Bacterial growth of a *Mtb* Δ*ppe51* deletion mutant in minimal medium with glucose and trehalose supplementation

**Figure 3:**
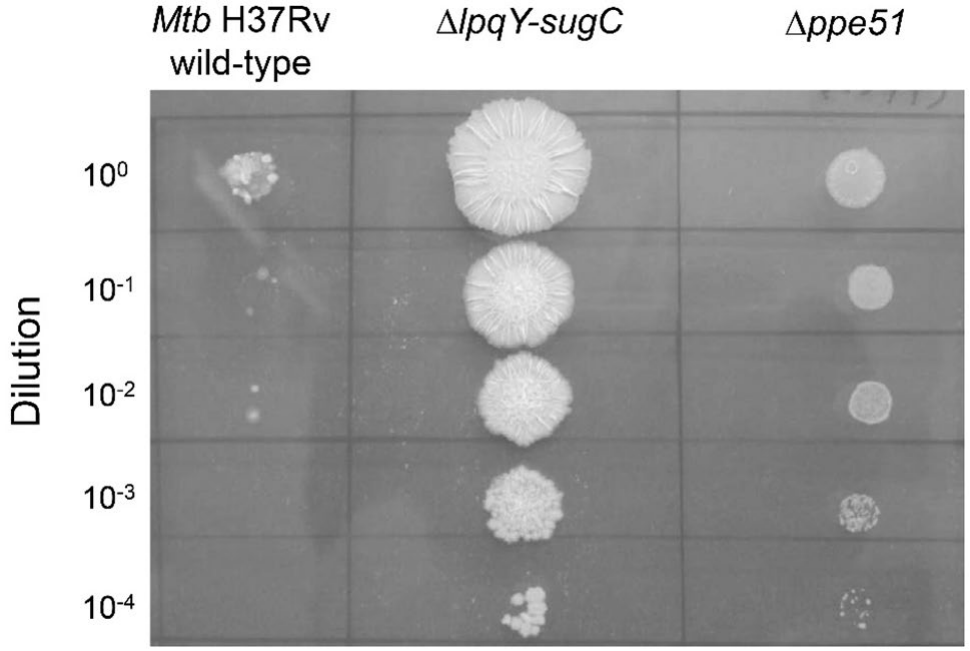
6-TreAz resistance of the *Mtb* Δ*ppe51* deletion mutant. 10 µl aliquots of tenfold-serially diluted cell suspensions of *Mtb* H37Rv wild-type, the Δ*lpqY-sugC* gene deletion mutant and the Δ*ppe51* gene deletion mutant were spotted onto Middlebrook 7H10 agar containing 1 mM 6-TreAz and incubated for 3 weeks at 37 °C. The Δ*ppe51* gene deletion mutant is unable to utilize glucose as will be demonstrated in Fig. 4 and therefore exhibits a growth defect on the used solid medium that contains glycerol and glucose as main carbon sources.

To further analyze the function of PPE51 in trehalose uptake, we generated a site-specific gene deletion mutant in *ppe51* (Rv3136) using the specialized transduction method. The Δ*ppe51* deletion strain was subjected to whole genome sequencing to confirm its genotype. In addition to the *ppe51* gene deletion, the sequencing also revealed a second-site non-synonymous mutation in the hypothetical gene Rv2662 (242 G→A, leading to amino acid substitution C81Y) and a base insertion in the mycocerosic acid synthase gene *mas* causing a frame shift (+ 1 bp insertion (+ a) at nucleotide position 5560 out of 6336 bp). This *mas* gene is responsible for the synthesis of multi-methyl branched mycocerosic acids, which form a segment of the phthiocerol dimycocerosate (PDIM) structure. PDIM loss is very common during in vitro culturing of *Mtb* strains^29^. Although growth at 1 mM 6-TreAz was weaker as compared to the Δ*lpqY-sugC* deletion strain, the Δ*ppe51* deletion mutant exhibited a clear resistance toward this azidosugar (Fig. 3), corroborating the phenotype of the spontaneous resistant mutants and proving the role of *ppe51* in resistance toward 6-TreAz.

**Figure 4:**
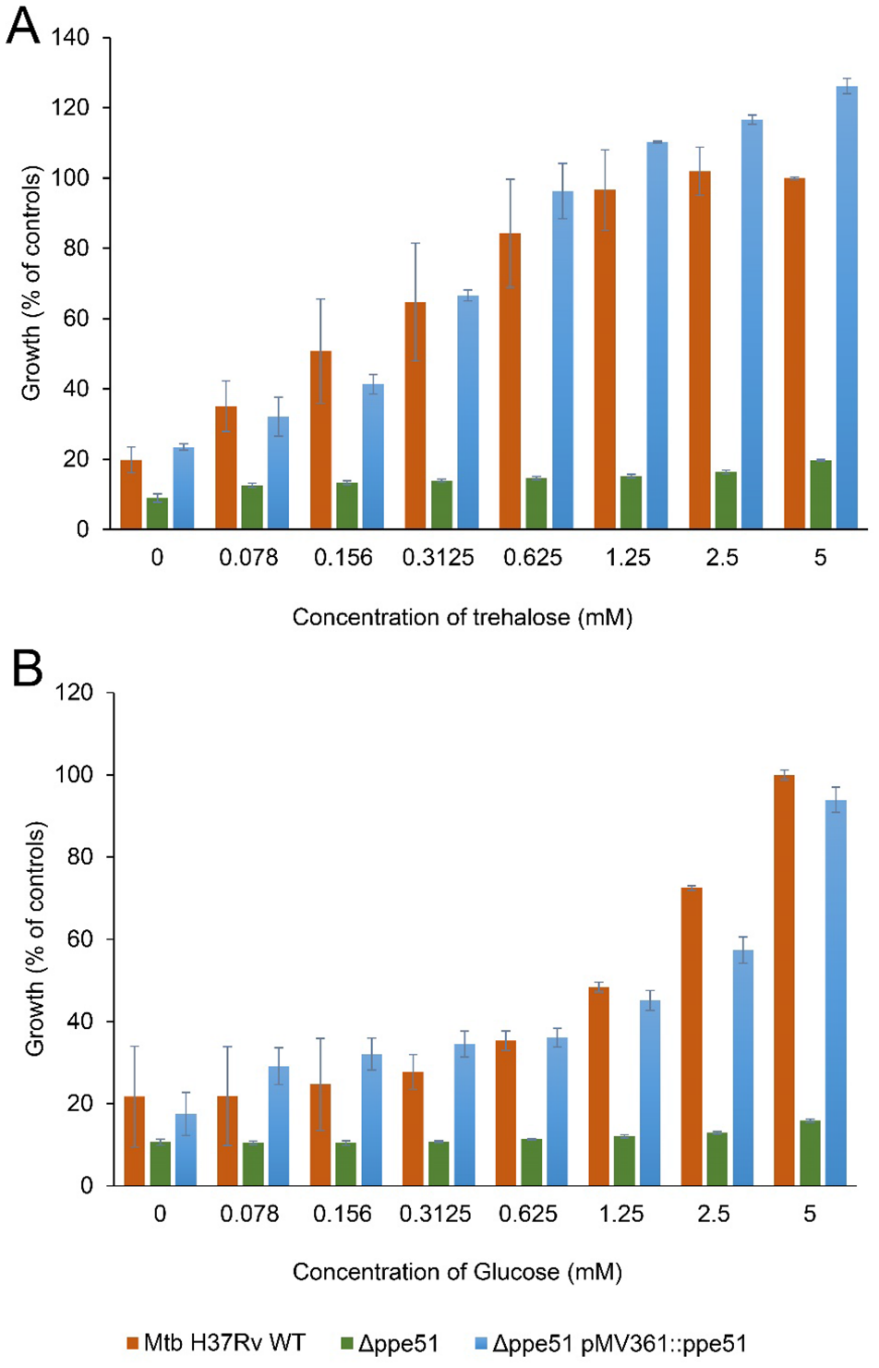
Growth of the *Mtb* Δ*ppe51* deletion mutant in minimal medium with glucose or trehalose as the sole carbon source. Cells of *Mtb* H37Rv wild-type (WT) (red bars), the Δ*ppe51* mutant (green bars) and the complemented mutant *Δppe51* pMV361::*ppe51* (blue bars) were cultivated in minimal medium containing increasing concentrations (0–5 mM) of trehalose (**A**) or glucose (**B**), respectively. Growth was determined using the resazurin microplate assay and normalized to cells of *Mtb* H37Rv wild-type grown in Middlebrook 7H9 complete medium (= 100% growth) or medium only control (= 0% growth). Values are means of triplicates ± SD. Growth defects of the Δ*ppe51* mutant observed at 5 mM concentration of both sugars compared to wild-type or the complemented mutant was statistically significant (*p* < 0.005 as determined using unpaired *t* test).

**Figure 5:**
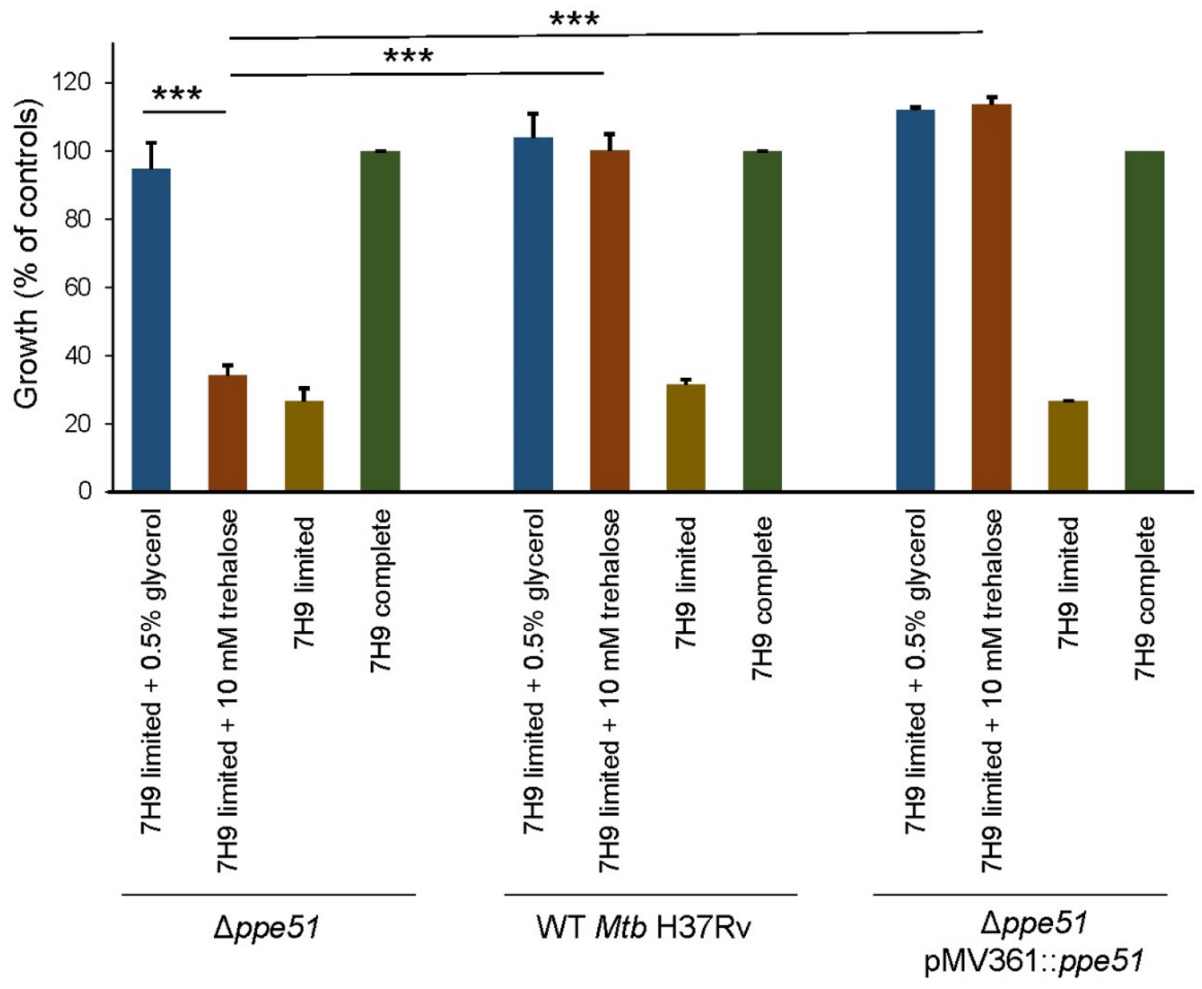
Growth of the *Mtb* Δ*ppe51* deletion mutant in 7H9 limited medium with glycerol or trehalose as the sole carbon source. Cells of *Mtb* H37Rv wild-type (WT), the Δ*ppe51* mutant and the complemented mutant *Δppe51* pMV361::*ppe51* were cultivated in 7H9 limited medium containing 0.5% (v/v) glycerol (blue bars), 10 mM trehalose (red bars) or no additional carbon source (brown bars). Strains grown in 7H9 complete medium served as positive controls (green bars). Growth was determined using the resazurin microplate assay and normalized to cells of *Mtb* H37Rv wild-type grown in 7H9 complete medium (= 100% growth) or medium only control (= 0% growth). Values are means of two independent experiments each performed in triplicates ± SD. Growth differences between the indicated groups were statistically significant (***p < 0.005 as determined using unpaired *t* test).

In addition to the Δ*ppe51* deletion strain, we also generated a corresponding complemented strain Δ*ppe51* pMV361::*ppe51* that constitutively expresses the wild-type *ppe51* gene from an integrative single-copy plasmid and could therefore compensate for the loss of transporter function. Next, we analyzed the growth of the Δ*ppe51*, Δ*ppe51* pMV361::*ppe51* and the H37Rv wild-type strain in minimal medium supplemented with increasing concentrations (0–5 mM) of trehalose or glucose to study the effect on growth of *Mtb*. The wild-type and the Δ*ppe51* pMV361::*ppe51* complemented strain showed a gradual increase in growth with increase in the concentrations of carbon sources provided. The Δ*ppe51* deletion strain was not able to grow on the minimal medium with or without supplementation of glucose or trehalose, which strongly suggests a proposed function of PPE51 as a transporter for both sugars (Fig. 4). In addition, we also measured growth of H37Rv wild-type, the Δ*ppe51* deletion strain and the Δ*ppe51* pMV361::*ppe51* complemented mutant in 7H9 medium + 0.05% tyloxapol (referred to as 7H9 limited medium) containing either trehalose or glycerol, respectively. Cultures grown in 7H9 + 0.05% tyloxapol medium supplemented with 10% ADS and 0.5% glycerol (referred to as 7H9 complete medium) were used as positive control. The Δ*ppe51* deletion strain did not grow in 7H9 limited medium containing trehalose but displayed normal growth in 7H9 complete medium. Complementation of this mutant restored growth similar to the wild-type strain (Fig. 5), demonstrating that the growth defect of the mutant was unequivocally attributed to the loss of *ppe51*. Previous studies have shown that a PDIM-proficient *ppe51* deletion strain was unable to utilize glycerol as a carbon source, whereas a PDIM-deficient *ppe51* deletion strain harboring a loss-of-function second-site mutation in *fadD26* could grow on both glucose and glycerol as the sole carbon source^22^. In contrast to glucose and trehalose, we observed that our Δ*ppe51* mutant was able to utilize glycerol as the sole carbon source when cultivated in 7H9 limited medium containing 0.5% glycerol (Fig. 5). This could result from an impaired formation or complete loss of PDIM due the observed spontaneous second-site frame shift mutation in the *mas* gene that might allow the Δ*ppe51* deletion mutant to use glycerol for growth in a PPE51-independent manner. However, no growth of the Δ*ppe51* deletion mutant was observed with trehalose as the sole carbon source under these conditions, indicating that trehalose was unable to enter the cells in a PPE51-independent manner despite the defect in PDIM biosynthesis.

### The Δ*ppe51* deletion mutant shows reduced fluorescence intensity when labelled with 6-TreAz and AF488 DIBO

**Figure 6:**
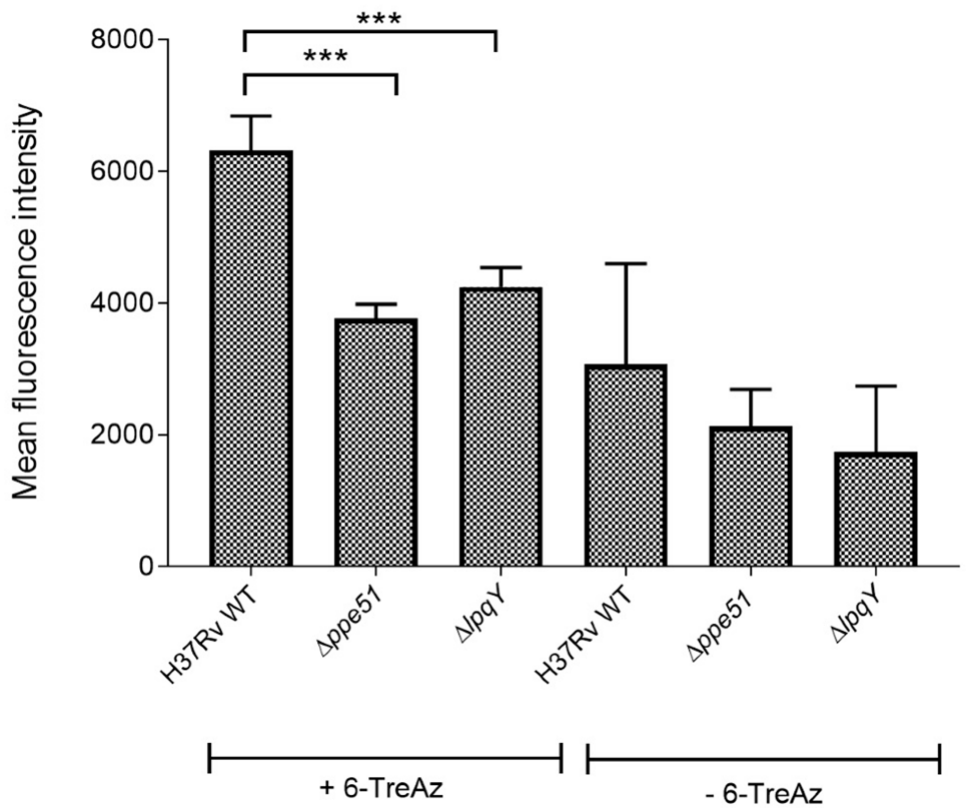
Metabolic labelling of *Mtb* strains employing click chemistry proves PPE51-dependent uptake of 6-TreAz. *Mtb* strains were incubated with 100 µM 6-TreAz for 3 days and subsequently conjugated with AF488 DIBO alkyne. Mean fluorescence intensity (MFI) was measured for 50,000 cells from each sample in each experiment. Data shown is the mean of three independent experiments ± SD. Differences in MFI between the indicated groups were statistically significant (***p < 0.005 as determined using unpaired *t* test). The high background in the controls that were not incubated with 6-TreAz is presumably due to non-specific association of the secondary DIBO-fluorophore.

*Mtb* strains are amenable to labeling with various trehalose analogs employing click chemistry to study the roles of different glycoproteins, membrane lipids and glycan molecules in the cellular envelope^13,30,31^. Copper-free click chemistry is based on the principle of bioorthogonal labelling which involves alkyne-azide cycloaddition. *Mtb* cells cultivated with azide-functionalized metabolic substrates can be conjugated to a cyclooctyne-functionalized probe such as dibenzocyclooctynes (DIBO). In this study, to corroborate the defect in uptake of trehalose and trehalose analogues, *Mtb* wild-type (positive control), the Δ*lpqY* (Rv1235) gene deletion mutant (negative control) and the Δ*ppe51* gene deletion mutant were grown in 7H9 medium containing 100 µM 6-TreAz and were subsequently labelled with Alexa Fluor 488 DIBO Alkyne. Mean fluorescence intensity (MFI) was measured for 50,000 cells via FACS. It was observed that the Δ*ppe51* mutant had reduced MFI compared to wild-type and almost similar MFI when compared to that of the Δ*lpqY* deletion strain indicating that uptake of 6-TreAz in wild-type is dependent both on PPE51 and LpqY-SugABC (Fig. 6). In the absence of PPE51 or LpqY, 6-TreAz could not enter the cytoplasm and hence AF488 DIBO Alkyne could not conjugate with azide molecules. The secreted antigen 85 complex working in reverse with 6-TreAz and TDM as substrates, however, can potentially produce some 6-TreAz-containing TMM extracellularly in the cell wall compartment. This 6-TreAz-containing TMM is incorporated into the cell wall and be conjugated with AF488 DIBO Alkyne, which may explain the background labeling even in the absence of uptake (Fig. 6).

### 6-TreAz resistant mutants show no cross-resistance against 2-TreAz

It was recently reported that not only 6-TreAz but also 2-azido trehalose (2-TreAz) exhibits moderate anti-mycobacterial activity against *M. smegmatis*^23^. Therefore, to test whether 2-TreAz also inhibits growth of *Mtb* H37Rv, we grew wild-type strain and our spontaneous resistant mutants on 7H10 solid medium containing up to 2 mM of 2-TreAz. However, we observed only partial growth inhibition even at the highest tested concentration, indicating that 2-TreAz has a much weaker effect on growth of *Mtb* compared to 6-TreAz. Importantly, although the effect of 2-TreAz was rather weak, the 6-TreAz-resistant mutants did not show any improved growth compared to the wild-type (Supplementary Fig. 3). This demonstrates that the observed mutations do not mediate cross-resistance to 2-TreAz. When spontaneous 6-TreAz-resistant mutants were tested for growth on trehalose as the sole carbon source, we found that all tested clones were still capable of utilizing this sugar (Supplementary Fig. 4). This demonstrates that despite the subtle differences in chemical structure, the observed mutations in PPE51 obviously specifically impair uptake of 6-TreAz but not of unmodified trehalose. Thus, it is possible that these mutations may also allow discrimination between 6-TreAz and 2-TreAz. The definitive role of PPE51 in uptake and the antibacterial activity of 2-TreAz could be interrogated in a Δ*ppe51* gene deletion mutant. Unfortunately, due to limited compound availability, we could not test susceptibility of the Δ*ppe51* gene deletion mutant towards 2-TreAz yet to definitively assess the role of PPE51 in uptake and the antibacterial activity of 2-TreAz.

## Discussion

Although the complete genome sequence of the *Mtb* wild-type strain H37Rv was revealed in 1998, many of its genes have not been fully functionally characterized yet. One such group of genes are the PE/PPE family of proteins. Initially, a set of PE/PPE proteins were known to contribute to the evasion of host immunity and replication in human macrophages^15^. It is very recently that these proteins gained attention in relevance to uptake of various molecules across the mycomembrane. In our study, we investigated growth inhibition of *Mtb* by the azide-functionalized sugar 6-TreAz. Inhibition of mycobacterial growth by 6-TreAz was previously observed with *Mycobacterium aurum*^14^. It was shown that this compound inhibited bacterial growth at 200 µg/ml on solid medium and also suppressed mycolyltransferase activity of the antigen 85 complex by 60% at a concentration of 100 µg/ml. It also resulted in lesser synthesis of TMMs and TDMs^14^. This compound also caused growth inhibition against planktonic cells of *M. smegmatis* at higher concentrations (500 µM) and displayed a significant anti-biofilm activity at 50 µM^23,24^. In the present study, we examined the anti-mycobacterial activity of the compound against *Mtb* H37Rv wild-type strain and observed growth inhibition at 1 mM. Although this activity is much too weak to provide a basis for antimycobacterial drug development, it offers a selectable tool for studying trehalose transport and metabolism.

Very recently, *Mtb* was shown to be inhibited by agrichemical compounds such as 3,3-bis-di(methyl sulfonyl) propionamide (3bMP1) and carbohydrate derivatives with thio group such as thio-glycoside. Spontaneous resistant mutants raised against these anti-mycobacterial compounds were characterized by whole genome sequencing and revealed PPE51 as a determinant of resistance. Subsequent biochemical characterization of PPE51 in these studies demonstrated that PPE51 is required for transport of low-molecular weight molecules such as the studied anti-mycobacterial compounds but also for carbohydrates such as glucose, glycerol, maltose and lactose^21,22^. However, information about the role of PPE51 in transport of trehalose was not available so far. Given the central importance of trehalose for viability of *Mtb*, it is important to understand the uptake of exogenous trehalose across the mycomembrane. In our study, we harnessed the antimycobacterial property of the analogue 6-TreAz as a surrogate to identify resistance determinants that might reveal genes involved in transport of trehalose across the mycomembrane in an unbiased manner. Mutations occurred in *ppe51*, *eccC5* and in genes involved in PDIM biosynthesis. Our experiments regarding growth on selected carbon sources, genetic complementation and click-chemistry labelling studies employing 6-TreAz and AF488DIBO clearly revealed that trehalose uptake across the mycomembrane is strictly mediated through PPE51, whereas the LpqY-SugABC ABC transporter is responsible for transport across the cytoplasm membrane.

The role of PDIM biosynthesis in relation to the essentiality of PPE51 was already observed by Wang et al. during their effort to generate a *ppe51* gene deletion in *Mtb*. They showed that the function of PPE51 is strictly essential for viability of *Mtb* during growth on carbohydrates unless loss of PDIM biosynthesis apparently enables the PPE51-independent uptake across the mycomembrane^22^. While a PDIM-proficient *ppe51* deletion strain was unable to utilize either glycerol or glucose as the sole carbon source, a PDIM-deficient *ppe51* deletion strain harboring a loss-of-function second-site mutation in *fadD26* could grow on both glucose and glycerol as the sole carbon source. In contrast, our Δ*ppe51* mutant harbouring a second-site frame shift mutation in the *mas* gene could not utilize trehalose and glucose but was able to grow on glycerol as the sole carbon source. This could result from different mutations (*fadD26* gene in Wang et al. vs. *mas* gene in our study) that might have affected PDIM biosynthesis to different degrees. We speculate that the frame shift mutation did not abolish *mas* gene function completely in our Δ*ppe51* mutant, leading to at least some residual PDIM formation that rendered the barrier function of the mycomembrane largely intact towards carbohydrates under the tested culture conditions, with exception of glycerol.

Korycka-Machała et al. reported that the uptake of other disaccharides such as maltose and lactose is also mediated through PPE51, and they also noticed that some isolated resistant mutants harbored mutations in *eccC5*, similar to our findings^21^. Recently, it has been demonstrated that EccC5 is part of the ESX-5 secretion apparatus^28^, and that numerous PE and PPE proteins are secreted in an ESX-5-dependent manner in *Mycobacterium marinum*^18^. Although PPE51 has not yet been formally established as a specific ESX-5 substrate, the findings by Korycka-Machała et al. and our own data suggest that the ESX-5 apparatus is involved in secretion of PPE51 and/or its proper translocation and insertion into the mycomembrane. The observed mutations in *eccC5* might impair secretion of protein substrates including PPE51 by the ESX-5 secretion apparatus, thus leading to the uptake defect that confers resistance towards 6-TreAz or thioglycosides.

**Figure 7:**
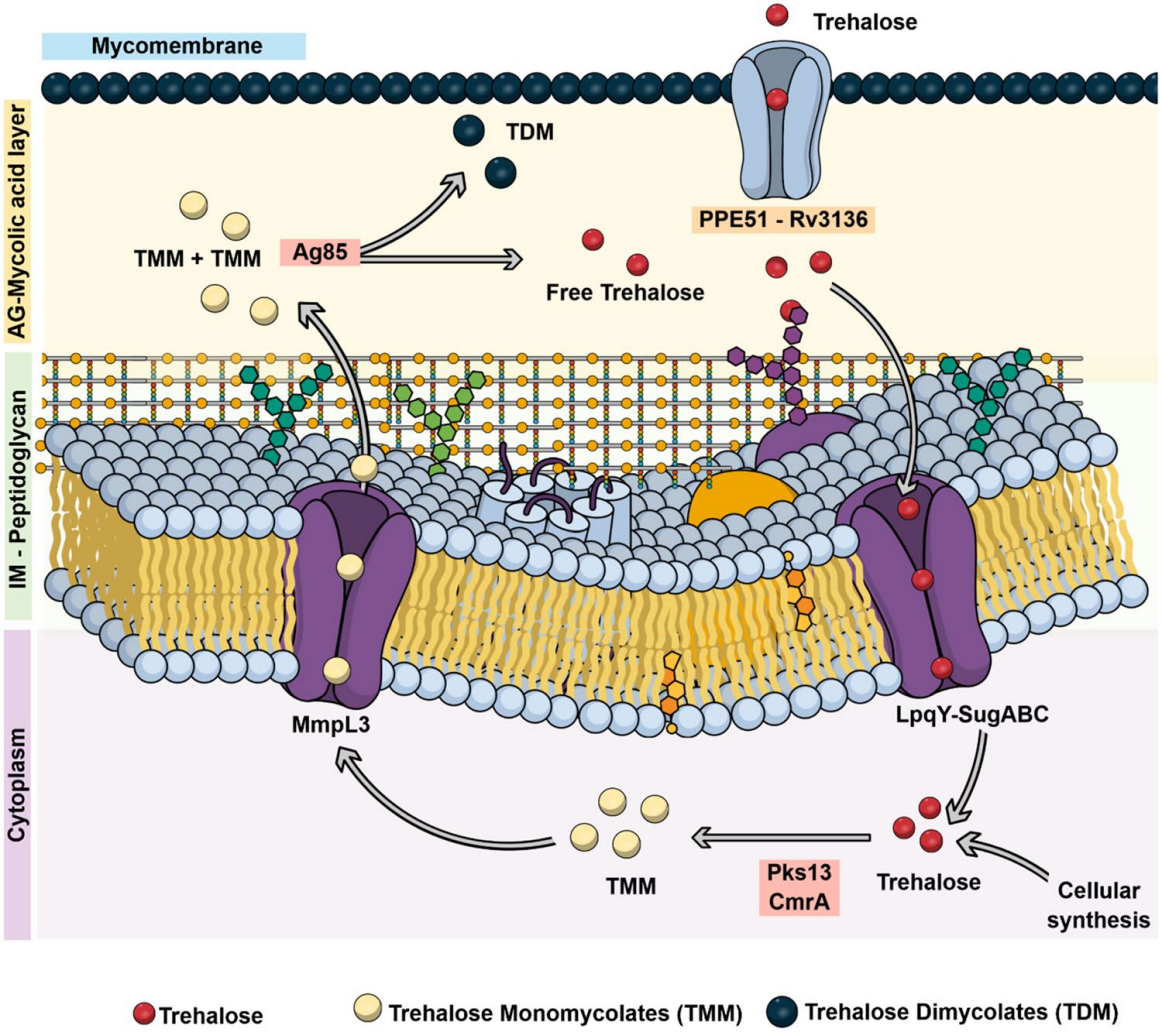
Simplified scheme summarizing transport and metabolism of trehalose and trehalose mycolates in *Mtb*. Exogenous trehalose is translocated across the mycomembrane via the PPE51 transporter. The LpqY-SugABC transporter allows the uptake of trehalose through the cytoplasmic membrane and recycles free trehalose that is released during synthesis of trehalose dimycolates (TDM) from trehalose monomycolates (TMM) by the mycoloyl transferases of the Antigen 85 complex (Ag85). The Ag85 also catalyzes the synthesis of arabinogalactan-mycolates from TMM, which is not depicted here for the sake of simplicity. Intracellularly, mycolates are conjugated to trehalose to yield TMM involving the enzymes Pks13 and CmrA, whereas the MmpL3 transporter mediates the secretion of TMM. *IM* inner membrane, *AG* arabinogalactan.

Taken together, our findings suggest that PPE51 serves as the gateway for the uptake of trehalose and trehalose analogs across the mycomembrane in addition to glucose, glycerol and other disaccharides. Figure 7 represents a simplified model of our current knowledge about the transport and metabolism of trehalose (analogues) in the different subcellular compartments of *Mtb*.

## Materials and methods

### Bacterial strains and growth conditions

All *Mtb* strains were grown at 37 °C either in Middlebrook 7H9 complete medium (Middlebrook 7H9 supplemented with 10% (v/v) ADS enrichment (5%, w/v, bovine serum albumin fraction V; 2%, w/v, glucose; 0.85%, w/v, sodium chloride), 0.5% (v/v) glycerol and 0.05% (v/v) tyloxapol) or on solidified 7H10 agar supplemented with 10% (v/v) ADS and 0.5% (v/v) glycerol unless otherwise stated. For the selection of appropriate strains, hygromycin (50 mg/l), apramycin (40 mg/l) and kanamycin (40 mg/l) were added. List of strains used for this study is provided in Supplementary Table 1. For testing growth on sole-carbon sources, *Mtb* strains were also grown in minimal medium [per liter: 0.15 g l-Asparagine × H_2_O, 0.5 g (NaH_4_)_2_SO_4_, 1 g KH_2_PO_4_, 2.5 g Na_2_HPO_4_, 50 mg ferric ammonium citrate, 0.5 g MgSO_4_ × 7 H_2_O, 0.5 mg CaCl_2_, 0.1 mg ZnSO_4_, 0.05% (v/v) tyloxapol, pH 7.0] and in Middlebrook 7H9 limited medium [containing only Middlebrook 7H9 and 0.05% (v/v) tyloxapol].

### Construction of a merodiploid strain and generation of targeted gene deletion in *Mtb*

A merodiploid strain with an additional copy of the trehalose recycling transporter LpqY–SugABC (Rv1235–1238) in the *Mtb* genome was generated by electroporating the integrative plasmid pMV306(Kan)::Rv1235–1238^11^ (1000 Ω, 25 µF, 2500 V) into *Mtb* H37Rv wild-type strain. This additional copy of the gene cluster compensates for any loss of Rv1235–1238 gene functions in spontaneous resistant mutants.

The *Mtb* Δ*ppe51* mutant was generated by allelic exchange using specialized transduction as described previously^32,33^. Briefly, the regions flanking *ppe51* (Rv3136) were amplified using Phusion DNA polymerase (New England Biolabs) using the primer pairs ppe51-LL-Van91I + ppe51-LR-Van91I and ppe51-RL-Van91I + ppe51-RR-Van91I (Supplementary Table 2), respectively, and cloned into an allelic exchange vector containing a hygromycin selection marker. The PacI digested vector was cloned into shuttle phasmid phAE159 and electroporated into *M. smegmatis* (1000 Ω, 25 µF, 2500 V)*.* The shuttle phasmid produces phages containing recombinant DNA in *M. smegmatis* at the permissible temperature of 30 °C. High-titer phage lysates were used to transduce *Mtb* cells for gene deletion and replacement by the hygromycin selection marker (Supplementary Fig. 5). Transductants were obtained on selection plates containing hygromycin (50 mg/l) after incubation for 3–4 weeks at the non-permissive temperature of 37  C.

### *Mtb* genomic DNA extraction and whole genome sequencing

The wild-type strain, spontaneous resistant mutants and the *ppe51* deletion mutant were cultured in 7H9 complete medium and the genomic DNA was extracted using CTAB-lysozyme method. Briefly, the cultures were pelleted and incubated with 9:1 mixture of GTE (50 mM glucose, 25 mM Tris–HCl, 10 mM EDTA) and lysozyme solution (10 mg/ml) overnight at 37˚C. Next day, 150 µl of 2:1 solution of 10% SDS and 10 mg/ml proteinase K was added and incubated for 30 min at 55 °C. Then, 200 µl of 5 M NaCl was added and mixed gently. 160 µl of preheated CTAB (cetrimide-hexadecyltrimethylammonium bromide in water) was added and mixed gently followed by incubation for 10 min at 65 °C. Then an equal volume of 24:1 (v/v) chloroform/isoamyl alcohol was added and mixed vigorously and then centrifuged for 5 min. The aqueous layer was then transferred to fresh microcentrifuge tube and the previous step was repeated to get rid of residual organic phase. 560 µl of 70% isopropanol was then added to the aqueous layer for precipitating the genomic DNA. This step was followed by incubation for 5 min and then centrifugation for 10 min. The pellet was then washed with 70% ethanol, air dried and resuspended in water.

Genomic DNA samples used for genome sequencing were quantified by photometric measurement using a NanoDrop One device (Thermo Fisher Scientific Inc.) and quality measured by capillary electrophoresis using the Fragment Analyzer and the ‘High Sensitivity genomic DNA Assay’ (Agilent Technologies, Inc.). Library preparation was performed according to manufacturer’s protocol using ‘TruePrep DNA Library Prep Kit V2 for Illumina (1 ng) (Vazyme Biotech Co.; Ltd). Libraries were normalized to 4 nM and pooled and subsequently sequenced at the BMFZ, Heinrich Heine University, on a MiSeq system (Illumina Inc) with a read setup of 2 × 251 bp. The reads were assembled using a comparative genome assembly method, using *Mtb* H37RvMA as a reference (GenBank accession GCA_000751615.1)^34^. Mean depth of coverage ranged from 69x to 114x.

### Genetic complementations

For complementation purposes, the genes *ppe51* and *eccC5* were amplified by PCR using the primer pairs ppe51-5′-PacI + ppe51-3′-HindIII and eccC5-5′-PacI + eccC5-3′-HindIII, respectively (Supplementary Table 2), and cloned into the integrative single-copy plasmid pMV361 containing an apramycin resistance marker providing constitutive heterologous gene expression from the HSP60 promoter. The plasmids were transformed into *Mtb* cells via electroporation (1000 Ω, 25 µF, 2500 V). Transformants were selected on agar plates containing apramycin (40 mg/l). During transformation into cells of spontaneous 6-TreAz-resistant mutants generated in the merodiploid Mtb H37Rv strain harbouring the integrative plasmid pMV306(Kan)::Rv1235–1238, the plasmid-encoded integrase mediates excision of the integrated plasmid pMV306(Kan)::Rv1235–1238 and replacement by pMV361(Apra)::*ppe51* or pMV361(Apra)::*eccC5*, respectively, during selection on apramycin and absence of kanamycin.

### Resazurin microplate assay (REMA) for growth quantification

Trehalose and glucose dependent growth of *Mtb* strains was quantified using the resazurin microplate assay. Cells were grown in liquid medium containing various concentrations of the indicated carbon sources in a total volume of 100 μl in 96-well round-bottom microtiter plates and incubated at 37 °C. After 5 days, resazurin solution (10 μl/well from 100 μg/ml stock, Sigma-Aldrich) was added to the cells and incubated for 16 h at room temperature for reduction of resazurin to resorufin by aerobic respiration of metabolically active cells. Next, *Mtb* cells were inactivated by incubation with formalin (10%, v/v, final concentration) for 30 min at room temperature. Subsequently, fluorescence was measured using a microplate reader (TECAN) (excitation 560 nm, emission 590 nm).

### Metabolic labelling of *Mtb*

All *Mtb* strains were grown in Middlebrook 7H9 complete medium until OD_600 nm_ ~ 0.8. 50 µl from the stock culture was added to 950 µl Middlebrook 7H9 complete medium containing 100 µM 6-TreAz in a 1.5 ml screw-cap tube and incubated for 3 days at 37 °C. After 3 days, the cells were centrifuged at 13,000 rpm for 5 min and the supernatant was discarded. The cells were then washed twice with PBS containing 0.5% (w/v) bovine serum albumin (PBSB) by centrifugation. After the washing step, the cells were incubated with 1 ml from 1:500 dilution of 1 mM Alexa Fluor 488 DIBO Alkyne stock solution in DMSO (ThermoFisher Scientific) at room temperature for 45 min in dark. Then, cells were washed again with PBSB twice and inactivated with 4% paraformaldehyde for 60 min at room temperature. The inactivated cells were washed with PBS and the final pellet was resuspended in 100 µl PBS. The cells were then prepared for measuring Mean fluorescence intensity (MFI) using flow cytometry. Flow cytometry was performed on BD LSRFortessa™ cell analyzer. Samples were gated according to forward and side scatter to exclude large cellular aggregates. MFI data was collected for 50,000 cells for each sample and processed using FlowJo (BD Biosciences).

## Supplementary Information

Supplementary Information.

## Data Availability

All data generated or analysed during this study are included in this published article and its Supplementary information files.
