# Supplementary material for: PPE51 mediates uptake of trehalose across the mycomembrane of Mycobacterium tuberculosis
Source: Sci Rep. 2022 Feb 8;12:2097. doi: 10.1038/s41598-022-06109-7 (PMC8826857; doi:10.1038/s41598-022-06109-7)
Supplement: Supplementary file 1 — Supplementary Information. [file 41598_2022_6109_MOESM1_ESM.pdf]

Supplementary Information

PPE51 mediates uptake of trehalose across the mycomembrane of *Mycobacterium tuberculosis*

Mohammed Rizwan Babu Sait[1,5], Hendrik Koliwer-Brandl[1,5], Jessica A. Stewart[2], Benjamin M. Swarts[2], Marc Jacobsen[3], Thomas R. Ioerger[4], Rainer Kalscheuer[1*]

[1] Institute of Pharmaceutical Biology and Biotechnology, Heinrich Heine University, 40225 Düsseldorf, Germany.

[2] Department of Chemistry and Biochemistry, Central Michigan University, Mount Pleasant, Michigan 48859, United States of America.

[3] Department of General Pediatrics, Neonatology, and Pediatric Cardiology, University Children's Hospital, Heinrich Heine University, 40225 Düsseldorf, Germany.

[4] Department of Computer Science, Texas A&M University, College Station, Texas 77843, United States of America.

[5] These authors contributed equally to this work.

***Correspondence:**

Rainer.Kalscheuer@hhu.de

Tel. +49 211 81 14180

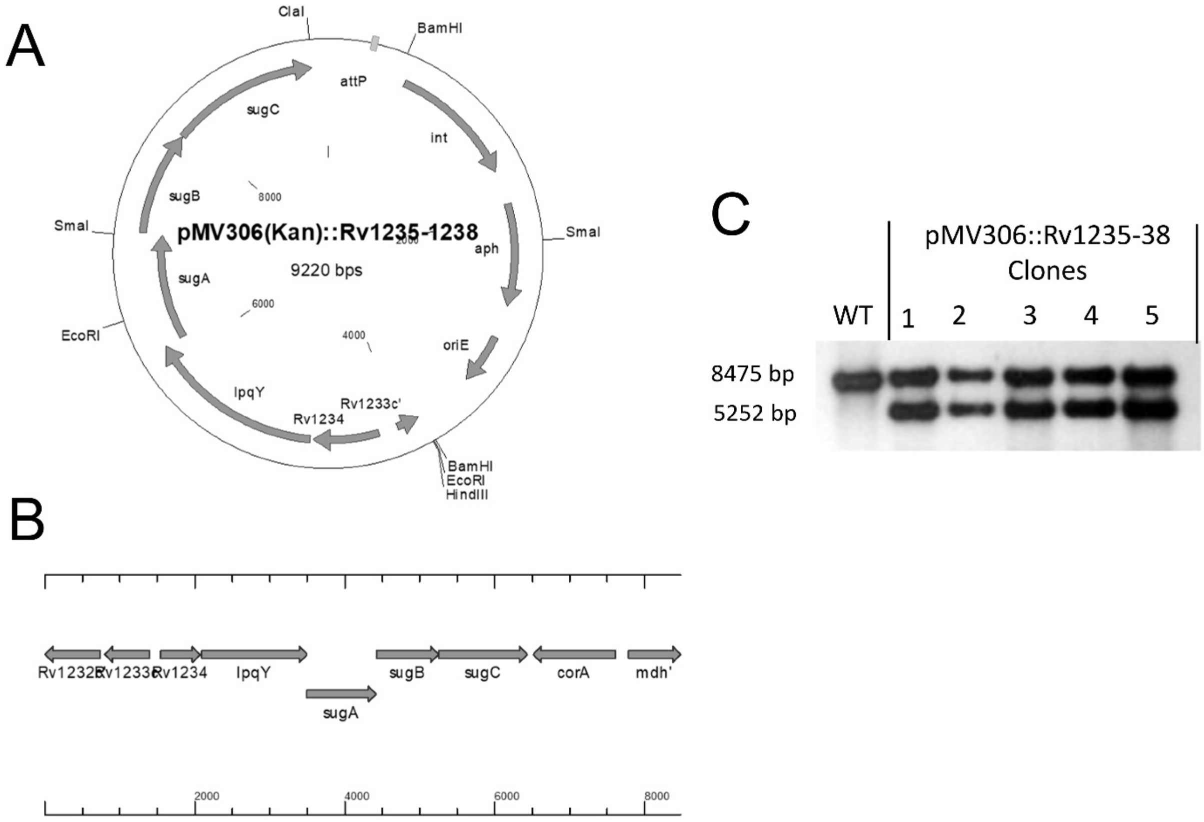

Supplementary Figure 1

Generation of a *lpqY-sugC* merodiploid *Mtb* H37Rv strain. (A) Map of integrating plasmid pMV306::Rv1235-1238. The plasmid-encoded integrase mediates single-copy integration of the construct into the *attB* site of the *Mtb* chromosome. The plasmid comprises the *lpqY-sugC* operon (Rv1235-1238) from the *Mtb* H37Rv plus the upstream region comprising the endogenous promoter. Restriction sites relevant for cloning or Southern analysis are indicated. aph, kanamycin resistance gene, int, integrase. (B) Molecular organization of the endogenous *lpqY-sugC* locus in *Mtb* H37Rv wild-type. Shown is a 8,475 bp *Bam*HI-*Cla*I fragment relevant for Southern analysis. (C) Southern blot proving presence of the additional copy of the *lpqY-sugC* operon in individual clones of merodiploid *Mtb* H37Rv. A *Sma*I-*Hin*dIII fragment obtained from plasmid pMV306::Rv1235-1238 containing the *lpqY-sugA* genes was used as the probe. The probe hybridized to a 8,475 bp fragment comprising the endogenous locus as shown in B and a 5,252 bp fragment comprising the merodiploid locus as shown in A employing *Bam*HI-*Cla*I double digested genomic DNA from *Mtb* H37Rv wild-type and individual clones of the merodiploid *lpqY-sugC Mtb* H37Rv strain. An uncropped image of this Southern blot is available in Supplementary Figure 6.

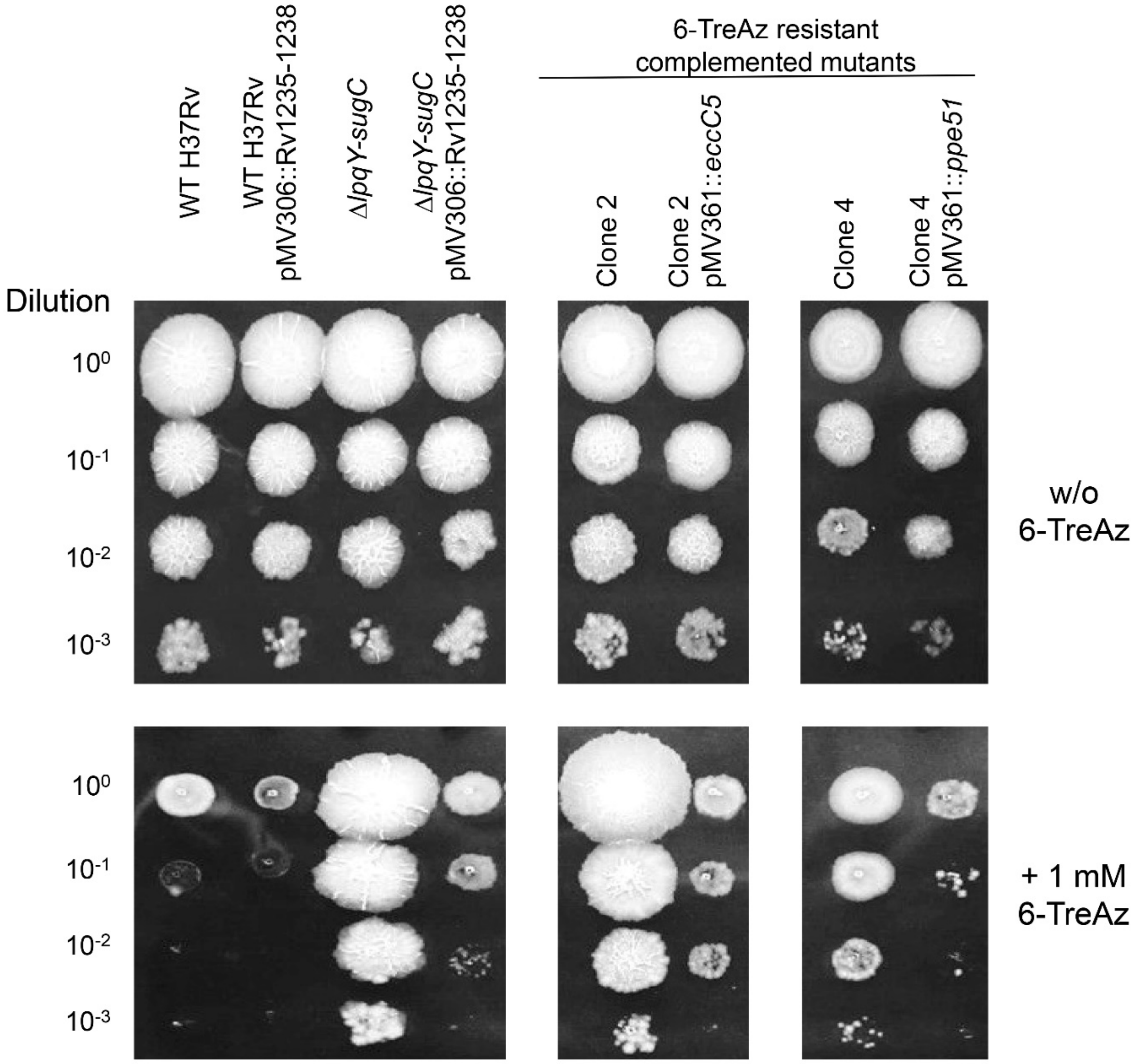

Supplementary Figure 2

Genetic complementation of spontaneous 6-azido trehalose (6-TreAz)-resistant *Mtb* mutants partially restores sensitivity towards 6-TreAz. 10 µl aliquots of tenfold-serially diluted cell suspensions of *Mtb* H37Rv wild-type (WT), the merodiploid strain *Mtb* H37Rv pMV306::Rv1235-1238, the Δ*lpqY-sugC* gene deletion mutant, the complemented mutant Δ*lpqY-sugC* pMV306::Rv1235-1238 and spontaneous 6-TreAz-resistant mutants complemented with *eccC5* or *ppe51*, respectively, from single-copy integrative plasmids were spotted each onto Middlebrook 7H10 agar containing 1 mM 6-TreAz or just solvent control (1% DMSO = w/o 6-TreAz). Plates were incubated for 3 weeks at 37°C. Sensitivity towards 6-TreAz was restored in the complemented spontaneous 6-TreAz-resistant mutants at least to the same extent as in the complemented mutant Δ*lpqY-sugC* pMV306::Rv1235-1238.

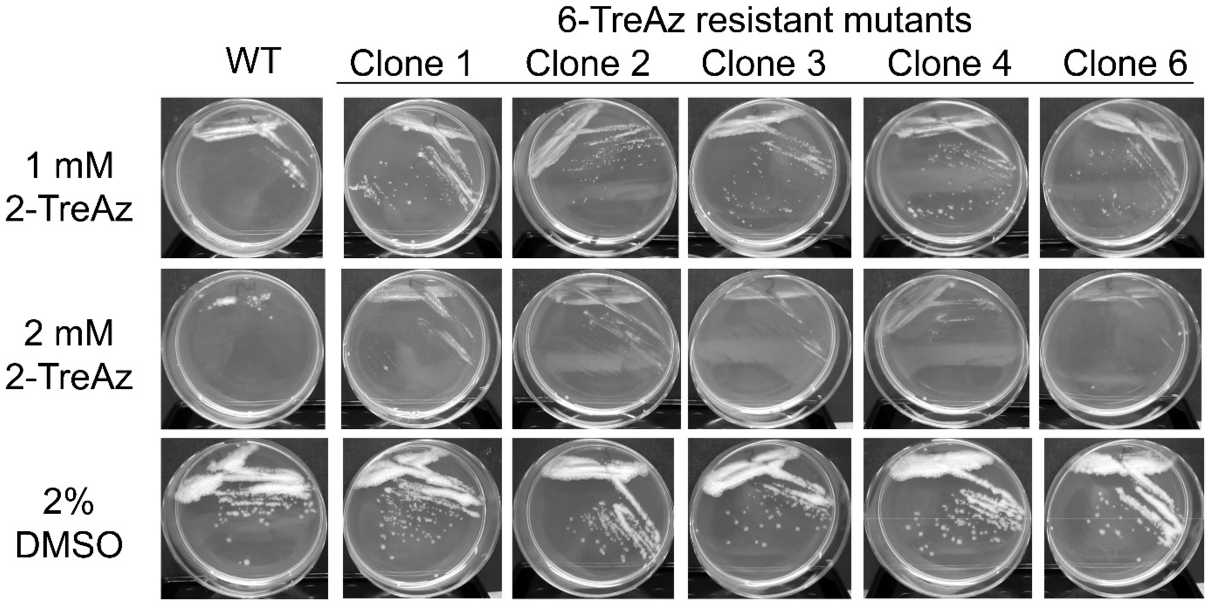

Supplementary Figure 3

Spontaneous 6-TreAz-resistant *Mtb* mutants are not cross-resistant towards 2-azido trehalose (2-TreAz). Cell suspensions of *Mtb* H37Rv wild-type (WT) and spontaneous 6-TreAz-resistant mutants were streaked out each onto Middlebrook 7H10 agar containing 1 mM and 2 mM 2-TreAz or just solvent control (2% DMSO). Plates were incubated for 3 weeks at 37°C. 2-TreAz at a concentration of 2 mM partially inhibited growth of *Mtb* H37Rv. Spontaneous 6-TreAz-resistant mutants exhibited no reduced sensitivity towards 2-TreAz.

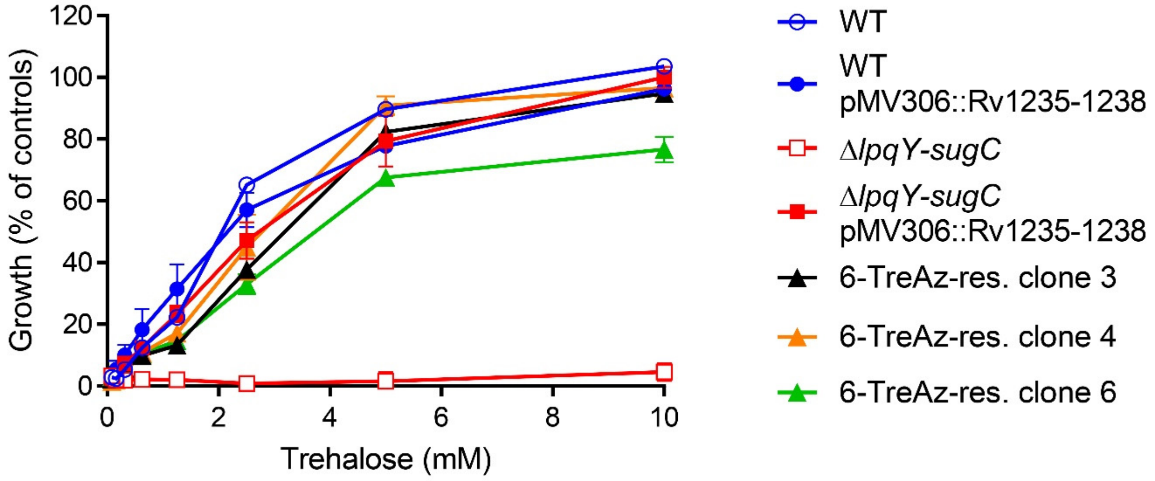

Supplementary Figure 4

Growth of spontaneous 6-TreAz-resistant *Mtb* mutants on trehalose as the sole carbon source. Cells of *Mtb* H37Rv wild-type (WT) (open blue circles), the *lpqY-sugC* merodiploid strain (WT pMV306::Rv1235-1238) (filled blue circles), the Δ*lpqY-sugC* gene deletion mutant (open red squares), the complemented mutant Δ*lpqY-sugC* pMV306::Rv1235-1238 (filled red squares), and spontaneous 6-TreAz-resistant mutant clones (black, orange and green triangles) were cultivated in minimal medium containing increasing concentrations (0 mM to 10 mM) of trehalose as the sole carbon source. Growth was determined using the resazurin microplate assay and normalized to cells of *Mtb* H37Rv wild-type grown in Middlebrook 7H9 complete medium (= 100% growth) or medium only control (= 0% growth). Values are means of triplicates ± SD.

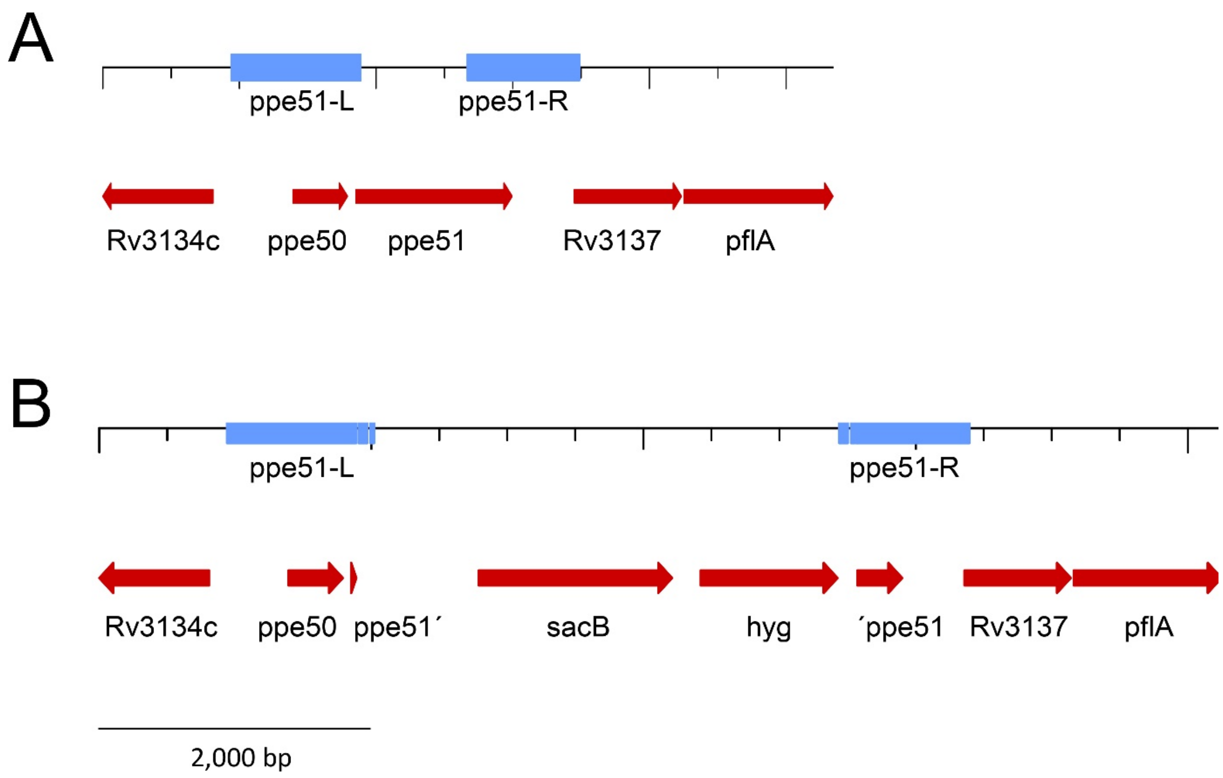

Supplementary Figure 5

Schematic organization of the *ppe51* gene locus in *Mtb* H37Rv wild-type (A) and the Δ*ppe51* gene deletion mutant (B). The positions of the allelic exchange substrates (ppe51-L, ppe51-R) used to generate the mutants are indicated. sacB, levansucrase gene, hyg, hygromycin resistance gene. The genotype of the Δ*ppe51* gene deletion mutant was verified by whole-genome sequencing as described in the main text.

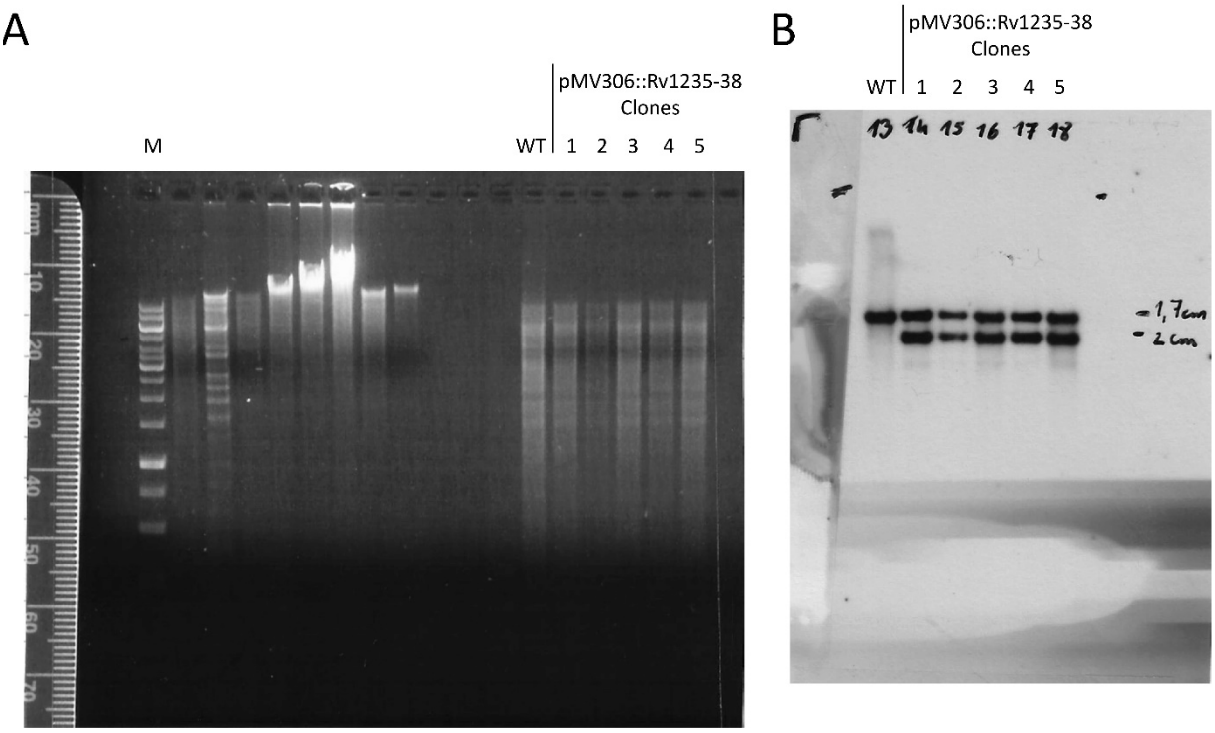

Supplementary Figure 6

Uncropped images of the agarose gel (A) and corresponding Southern blot (B) related to Supplementary Figure 1C. For detail, see legend to Supplementary Figure 1C. M, 1kb DNA size ladder.

Supplementary Table 1. List of *Mtb* strains used in the study.

Mtb strains	Antibiotic Resistance	Source /Reference
Mtb H37Rv wild-type	-	lab strain
Mtb Δ*lpqY-sugC* (Rv1235-1238)	Hygromycin	Kalscheuer et al., 2010
Mtb Δ*lpqY-sugC* pMV306::1235-1238	Hygromycin, Kanamycin	Kalscheuer et al., 2010
Mtb H37Rv pMV306::Rv1235-1238	Kanamycin	This study
Mtb H37Rv pMV306::Rv1235-1238 6-TreAz resistant mutant clones 1 - 6	Kanamycin	This study
Mtb 6-TreAz-resistant mutant clone 2 pMV361::*eccC5*	Apramycin	This study
Mtb 6-TreAz-resistant mutant clone 4 pMV361::*ppe51*	Apramycin	This study
Mtb Δ*ppe51* (Rv3136)	Hygromycin	This study
Mtb Δ*ppe51* pMV361::*ppe51*	Hygromycin, Apramycin	This study

Reference: Kalscheuer R, Weinrick B, Veeraraghavan U, Besra GS, Jacobs WR, Jr. Trehalose-recycling ABC transporter LpqY-SugA-SugB-SugC is essential for virulence of *Mycobacterium tuberculosis*. Proc Natl Acad Sci U S A 107, 21761-21766 (2010).

Supplementary Table 2. List of primers used in the study. Relevant restriction sites used for cloning purposes are underlined.

Primer Name	Primer Sequence
ppe51-LL-Van91I	5' TTTTTCCATAAATTGGACCCGTCAGGGCGAGAATGAATC 3'
ppe51-LR-Van91I	5' TTTTTCCATTTCTTGGCGGAGTTGACTTCCGGTGGTAACAG 3'
ppe51-RL-Van91I	5' TTTTTCCATAGATTGGAGTTGGTTTCCGCGACCAGTCCC 3'
ppe51-RR-Van91I	5' TTTTTCCATCTTTTGGGCCAGAGCCAGCGCAAGCATTAG 3'
ppe51-5´-PacI	5' TTTTTTTAATTAAATGGATTTCGCACTGTTACCACC 3'
ppe51-3´-HindIII	5' TTTTTAAGCTTTTACCCTGCCGCGGGTGGGTGGG 3'
eccC5-5´-PacI	5´ TTTTTTTAATTAAATGAAGCGTGGTTTTGCCCGCCC 3´
eccC5-3´-HindIII	5´ TTTTTAAGCTTCTACCGACGCACCTCGGTGGCTG 3´
